# Localization of Lesions in Autoimmune Blistering Diseases Is Independent of Site-Specific Target Antigen Expression

**DOI:** 10.3390/life15020218

**Published:** 2025-01-31

**Authors:** Tina Rastegar Lari, Louis Macias, Lara Robrahn, Hasan Onur Dikmen, Jasper Prüßmann, Charlotte Kiehne, Simon Engster, Imke Weyers, Silke Szymczak, Nina van Beek, Markus H. Hoffmann, Enno Schmidt, Shirin Emtenani

**Affiliations:** 1 Lübeck Institute of Experimental Dermatology, University of Lübeck, 23562 Lübeck, Germany; tina.rastegarlari@uksh.de (T.R.L.); hasan-onur.dikmen@charite.de (H.O.D.); charlotte.kiehne@uksh.de (C.K.); simon.engster@gmail.com (S.E.); enno.schmidt@uksh.de (E.S.); 2 Institute of Medical Biometry and Statistics, University of Lübeck, 23562 Lübeck, Germany; louis.macias@uni-luebeck.de (L.M.); silke.szymczak@uni-luebeck.de (S.S.); 3 Department of Dermatology, Allergology and Venerology, University of Lübeck, 23562 Lübeck, Germany; lara.robrahn@student.uni-luebeck.de (L.R.); jasper.pruessmann@uksh.de (J.P.); nina.vanbeek@uksh.de (N.v.B.); markus.hoffmann@uni-luebeck.de (M.H.H.); 4 Institute for Systemic Inflammation Research, University of Lübeck, 23562 Lübeck, Germany; 5 Institute of Anatomy, University of Lübeck, 23562 Lübeck, Germany; imke.weyers@uni-luebeck.de

**Keywords:** autoimmune blistering disease, bullous pemphigoid, pemphigus, predilection sites, antigen expression

## Abstract

Autoimmune blistering diseases (AIBDs) involve autoantibodies targeting proteins in the epidermal/epithelial desmosome (pemphigus) or basement membrane zone (pemphigoid). Despite widespread antigen distribution, lesions exhibit a scattered involvement pattern. This study maps the frequency/severity of AIBD lesions on various body parts and investigates whether differential antigen expression contributes to specific predilection sites. We analyzed affected sites presenting blisters/erosions, erythematous/urticarial lesions, and mucosal lesions in bullous pemphigoid (BP-cohort 1, *n* = 65; BP-cohort 2, *n* = 119), pemphigus vulgaris (PV, *n* = 67), and pemphigus foliaceus (PF, *n* = 20) patients. To assess antigen expression, we conducted indirect immunofluorescence (IF) staining of 11 AIBD antigens from 13 anatomical sites of 10 body donors without AIBD. In BP, blisters/erosions and erythematous/urticarial lesions predominantly affected arms and legs, while PV/PF patients exhibited frequent involvement of buccal mucosa and back, respectively. IF staining identified significant regional differences in BP180, BP230, and integrin β4 expression, although these variations did not correlate with a higher lesion frequency/severity. Other antigens showed consistent expression across all regions. Our findings suggest that predilection sites for BP and PV/PF are largely unaffected by regional variations in antigen expression but may be influenced by factors like microbiota, mechanical stress, sunlight exposure, local immunity, or genetics.

## 1. Introduction

Autoimmune blistering diseases (AIBDs) are a paradigm of organ-specific, autoantibody-mediated autoimmune diseases that can be classified into two groups based on the location of split formation: intraepidermal blistering in the pemphigus group and subepidermal blistering in the pemphigoid group and dermatitis herpetiformis [1]. In pemphigus, autoantibodies target desmosomal cadherins, desmoglein (Dsg) 1 and Dsg3, which disrupt epithelial cell–cell adhesion, leading to intraepithelial blisters and erosions of the skin and the surface-close mucous membranes [2,3]. In contrast, pemphigoid diseases are characterized by autoantibodies against structural proteins at the dermal–epidermal junction [4,5].

Bullous pemphigoid (BP), the most common AIBD, is caused by autoantibodies against two hemidesmosomal proteins, BP180 (BPAG2, type XVII collagen) and BP230 (BPAG1, dystonin) [6]. In epidermolysis bullosa acquisita, autoantibodies target type VII collagen, while mucous membrane pemphigoid is distinguished by predominant mucosal involvement and autoantibodies against BP180, laminin 332, type VII collagen, BP230, and α6β4 integrin [7].

A key, yet unresolved, question in AIBD research concerns the distribution of cutaneous and/or mucosal lesions, which tend to appear in specific anatomical regions with varying frequency and severity. One possible explanation is that these regions exhibit higher levels of the target antigens, making them more susceptible to blister formation. This hypothesis is supported by findings showing that human mucosal keratinocytes express high levels of pemphigus vulgaris (PV) antigens but lower levels of pemphigus foliaceus (PF) antigens [8]. By examining the expression levels of AIBD antigens, we aim to understand how antigen abundance or scarcity, before disease onset, may contribute to lesion distribution in AIBD.

To investigate this hypothesis, we systematically examined the predilection sites of clinical lesions in patients with BP, PV, and PF. Additionally, we semi-quantitatively assessed the expression levels of key AIBD target antigens, including Dsg1, Dsg3, BP180, BP230, collagen VII, laminin α3, laminin β3, integrin α6, integrin β4, plectin, and cytokeratin 14, across 13 anatomical regions, aiming to identify potential correlations between antigen expression levels and lesion distribution.

## 2. Materials and Methods

### 2.1. Human Material

The study included the following cohorts of patients with AIBD: (i) BP cohort 1 (*n* = 65; females [56.9%], males [43.1%]; mean age, 79.9 years; median age, 83 years), (ii) BP cohort 2 (*n* = 119; 49 females (41%), 70 males (59%); mean age, 75 years; median age, 77 years), (iii) PV cohort (*n* = 67; females [56.2%], males [43.8%]; mean age, 65.6 years; median age, 55 years), and (iv) PF cohort (*n* = 20; females [38.8%], males [61.2%]; mean age, 66.9 years; median age, 71 years).

BP patients were diagnosed according to the following diagnostic criteria: (i) a compatible clinical picture without predominant mucosal involvement, (ii) positive immunoglobulin G (IgG) staining on the epidermal side of 1 M human salt-split skin using indirect immunofluorescence (IF) microscopy, and (iii) presence of circulating IgG autoantibodies against BP180-NC16A or BP230 using enzyme-linked immunosorbent assay (ELISA) (Euroimmun, Lübeck, Germany), and/or linear IgG and/or C3c deposition along the basement membrane zone (BMZ) through direct IF microscopy of perilesional skin biopsies [9,10]. Disease activity at initial diagnosis was assessed using the Bullous Pemphigoid Disease Activity Index (BPDAI) [11], which differentiates between blisters/erosions and erythematous/urticarial lesions.

Pemphigus was diagnosed based on the clinical picture, intercellular IgG binding to the epithelium of monkey esophagus, and IgG reactivity to Dsg1 and/or Dsg3, as measured using ELISA (Euroimmun) [3,12]. Disease severity was assessed using the Pemphigus Disease Area Index (PDAI) scoring system [13], which distinguishes between the following: (i) cutaneous activity (i.e., blisters, erosions, and new erythema), (ii) damage (i.e., post-inflammatory hyperpigmentation and erythema from resolving lesions), and (iii) mucosal lesions. Both BPDAI and PDAI scores range from 0 to 10 based on severity. All patients were newly diagnosed and had not received systemic immunosuppressive treatment prior to inclusion.

In cohorts 1, 3, and 4, site involvement was determined using BPDAI and PDAI data from clinical records. In cohort 2, lesions were recorded retrospectively from clinical photographs routinely taken between January 2012 and September 2023. For patients with multiple time points, only images from the first visit were analyzed. As the scores from both arms and both legs were identical in the blister and urticaria categories of BPDAI with the exception of two patients, a single score for arms and legs was used, taking the lowest score from either side. BPDAI mucosal lesions or regions within the mouth were combined into a single mouth score. Ordinal scores for both PDAI and BPDAI were categorized as absent, between 1 and 3, and above 3.

For analysis of antigen expression, skin and mucosal punch biopsies were obtained from 10 body donors (4 males and 6 females, all > 70 years old) with no history of AIBD. From each body donor, 5 mm postmortem biopsies were taken from 13 anatomical sites, including buccal mucosa, lower labial mucosa, lower conjunctiva, cheek, central sweating line of the breast, central sweating line of the back, flank at umbilical level, medial upper leg, dorsal lower leg, sole, medial upper arm, medial forearm, and palm. Tissue samples were embedded in Tissue-Tek O.C.T. compound (Sakura Finetek, Staufen, Germany) for IF examination.

### 2.2. Immunofluorescence Studies

IF staining was performed to semi-quantitatively assess the expression of key AIBD antigens, including Dsg1, Dsg3, BP180, BP230, collagen VII, laminin α3, laminin β4, integrin α6, integrin β4, plectin, and cytokeratin 14, across 13 different body sites. Tissue sections, 6 µm thick, were air-dried and incubated with serial dilutions of primary antibodies in phosphate-buffered saline (PBS) for 1 h at room temperature (RT). Isotype controls served as negative controls. Following washing with PBS, sections were incubated with secondary antibodies for 1 h at RT, washed, and mounted with DAPI (4′,6-diamidino-2-phenylindole) Fluoromount-G^®^ (SouthernBiotech, Birmingham, AL, USA). The stained sections were visualized using a Keyence microscope (BZ-9000E series, Keyence GmbH, Neu-Isenburg, Germany). Endpoint titers for each antibody were defined as the highest dilution showing positive staining. A detailed list of the primary and secondary antibodies used is available in Table S1.

### 2.3. Statistics

The assessment of differences in AIBD antigen expression across body sites in body donors was performed in two stages. First, for each of the 11 AIBD antigens, the global null hypothesis of no differences across all sites was tested using the non-parametric Friedman test. This test was applied in the case of paired samples to take into account that measurements from different sites were measured in the same individuals. Global *p*-values were adjusted using the Holm method [14]. For AIBD antigens with adjusted *p*-values <0.05, post hoc pairwise comparisons of sites were performed using a permutation-based null distribution, ensuring that *p*-values were adjusted for many pairs.

To compare the different components of the BPDAI and PDAI scores across body sites, the same approach was used for continuous components, including skin and mucosal erosion/blisters in BPDAI and PDAI, as well as urticaria in BPDAI. As the score frequency distribution by site was similar in both BPDAI cohorts, these were analyzed together. However, for dichotomous components such as the pigmentation score, Cochran’s Q test and McNemar test for paired samples were used to test the global and pairwise null hypotheses [15]. As before, global *p*-values were adjusted using the Holm method, and pairwise *p*-values were determined by a permutation-based null distribution that accounted for the number of pairs comparisons.

It was not feasible to perform a standard correlation analysis to correlate antibody titer measurements and BPDAI/PDAI scores, since these were measured in different individuals. Instead, the body site was used as an observational unit for this analysis. Mean antibody titers and median clinical scores across individuals at each site were calculated, and the correlation was estimated using Spearman’s rank correlation coefficient, with *p* values estimated using Monte Carlo resampling.

All statistical analyses were conducted using R (version 4.1, 2021, The R Foundation for Statistical Computing) using the R packages rstatix (https://CRAN.R-project.org/package=rstatix) [accessed on 17 April 2023] (functions friedman_test, cochran_test, and pairwise_mcnemar_test) and coin (functions symmetry test and spearman_test) [16].

## 3. Results

### 3.1. Arms and Legs Are the Most Common Sites for Blisters/Erosions and Erythematous/Urticarial Lesions in BP

**Figure 1:**
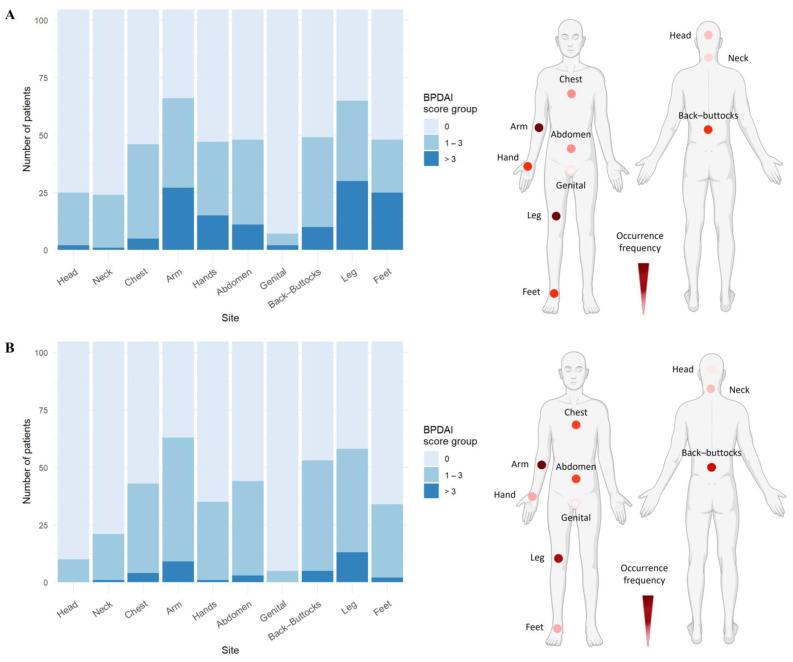
Skin blisters/erosions and erythematous/urticarial lesions occur predominantly on arms and legs in bullous pemphigoid patients. (**A**) Arms and legs were the most frequently and severely affected sites by skin blisters/erosions, followed by back/buttocks, abdomen, feet, hands, and chest. (**B**) Erythematous/urticarial lesions were predominantly observed on arms, followed by legs and trunk. Data include both BP cohorts (*n* = 105). Representative body models depict the frequency distribution of skin or urticarial lesions based on the number of patients showing BPDAI > 0, with darker colors indicating higher frequency (created with BioRender.com).

In BP cohort 1, all patients presented with cutaneous blisters/erosions, with arms (53.8%) and legs (50.7%) being the most frequently affected areas, followed by abdomen, back/buttocks, feet, hands, and chest (Figure 1A, Table S2). Notably, the most severe blisters/erosions also occurred on arms, legs, and feet. Additionally, a substantial proportion of patients had erythematous/urticarial lesions on their arms (53.8%, score 1), followed by legs, back/buttocks, abdomen, and chest. These lesions rarely reached a severity score greater than two (Figure 1B, Table S3).

Mucosal involvement was observed in 10.8% of patients in BP cohort 1, although no mucosal region exceeded a BPDAI score of two (Table S4). Buccal mucosa (7.7%) and soft palate (4.6%) were the most commonly affected mucosal sites. One patient had involvement of the nose, hard palate, lower gingiva, and anogenital region, with a clinical score of two, in addition to extensive skin lesions. Notably, no lesions were detected in eyes, floor of the mouth, or posterior pharynx. Pigmentation was noted in 47.7% of patients, predominantly on arms and legs (36.9% each) (Table S5).

These findings were consistent with BP cohort 2, where arms (77%) and legs (75.8%) were again the most common sites for blistering/erosions, followed by back/buttocks, feet, hands, and chest (Figure 1A, Table S2). Similarly, legs, arms, and feet were the most severely affected areas. The severity and frequency of erythematous/urticarial lesions (Figure 1B, Table S3) and the pattern of mucosal involvement (Table S4) closely mirrored the observations from BP cohort 1.

### 3.2. Trunk and Buccal Mucosa Are Predilection Sites in PV

**Figure 2:**
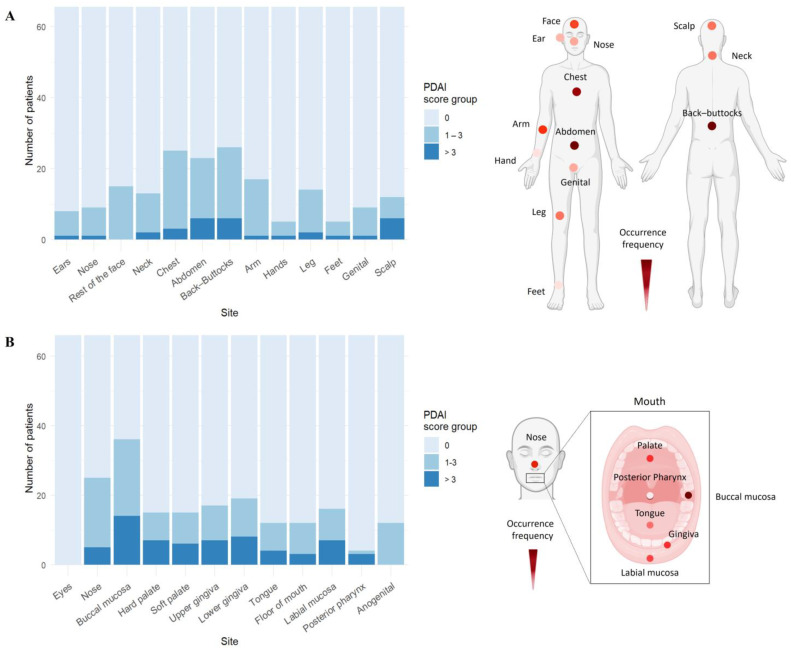
Trunk and buccal mucosa are the most frequently and severely affected sites in pemphigus vulgaris. (**A**) In PV patients (*n* = 67), the trunk area, including back/buttocks, chest, and abdomen, was the most affected by skin blisters. (**B**) Mucosal involvement was predominantly seen in buccal mucosa and gingiva, with additional lesions in the nasal and genital areas. Representative body models display the distribution frequency of skin or mucosal lesions based on the number of patients showing PDAI > 0 in the PV cohort, with darker colors signifying higher frequencies (created with BioRender.com).

In PV, mucosal lesions primarily affected the buccal mucosa (53.7%), while eyes were not affected (Figure 2B, Table S6). For cutaneous blisters/erosions, the most common and severely affected areas were back/buttocks (38.8%), chest (37.3%), abdomen (37.3%), arms (25.4%), and face (22.4%) (Figure 2A, Table S7). As anticipated, no ocular involvement was found in PV patients.

### 3.3. Trunk and Face Are Predilection Sites for Skin Blistering in PF

**Figure 3:**
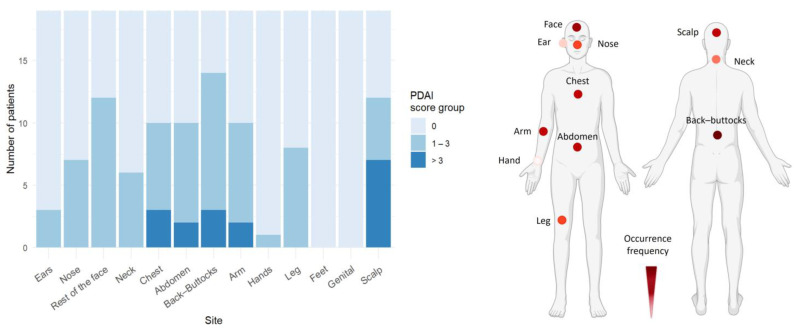
Pemphigus foliaceus skin lesions mainly affect trunk and face. In the PF cohort (*n* = 20), the trunk and face were the primary areas affected by skin lesions. Representative body models illustrate the frequency of skin lesion distribution based on the number of patients showing PDAI > 0 in the PF cohort, with darker colors representing higher frequencies (created with BioRender.com).

In the PF cohort, skin blistering predominantly affected the back/buttocks, face, abdomen, and chest (Figure 3, Table S7). As expected, no mucosal involvement was observed in PF.

### 3.4. AIBD Antigen Expression Shows Slight Variations in Different Skin and Mucosal Regions

**Figure 4:**
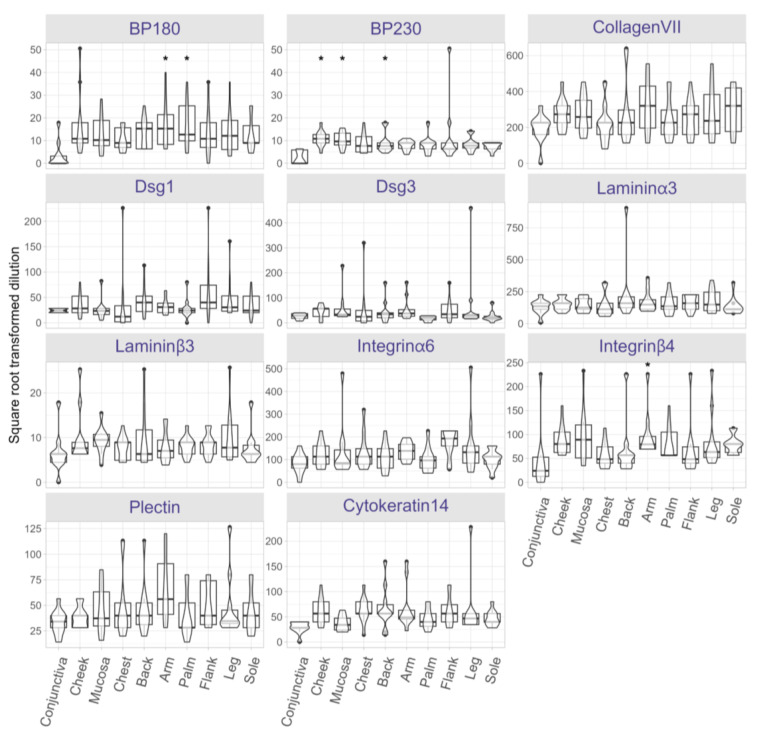
Protein expression landscape reveals slight variations in AIBD antigen expression across skin and mucosal regions. Protein expression of AIBD antigens was assessed in skin and mucosal tissue sections using serial dilutions of BP180, BP230, collagen VII, desmoglein (Dsg) 1, Dsg3, laminin α3, laminin β3, integrin α6, and integrin β4, plectin, and cytokeratin 14. Notable differences include significantly higher BP180 expression in skin biopsies from arms (*p* = 0.0003) and palms (*p* = 0.0007) compared to conjunctiva, which had the lowest expression. BP230 expression was significantly higher in the cheek (*p* = 0.00002), oral mucosa (*p* = 0.00004), and the back (*p* = 0.0091), relative to conjunctiva. Likewise, integrin β4 expression was significantly higher in arms (*p* = 0.0007) versus conjunctiva. Other antigens displayed no significant regional differences. Significant expression differences are marked with an asterisk.

To evaluate AIBD antigen expression across clinical predilection sites, antigen distribution was semi-quantified on skin and mucosal tissues obtained from 13 distinct anatomical sites of ten post-mortem body donors. The endpoint titer (i.e., highest titer) for each antibody was determined at each body site (Figure S1). Following correction for multiple comparisons using the Holm method, statistically significant differences (adjusted *p* < 0.05) were found for BP230, BP180, and integrin β4. Across all sites, BP180 expression was significantly higher in skin biopsies from arms (*p* = 0.0003) and palms (*p* = 0.0007) compared to conjunctiva (Figure 4). BP230 expression was more abundant in the cheek (*p* = 0.00002), oral mucosa (*p* = 0.00004), and the back (*p* = 0.0091), relative to conjunctiva. Additionally, integrin β4 expression was significantly higher in arms (*p* = 0.0007) compared to conjunctiva. No significant differences in the expression of other antigens were found between the body sites.

### 3.5. Expression Levels of AIBD Antigens Do Not Correlate with Clinical Scores

Lastly, we explored the correlation between antigen expression and clinical disease severity scores. We compared BP180, BP230, and integrin β4 expression in regions corresponding to BPDAI and PDAI scoring sites (e.g., conjunctiva, cheek, oral mucosa, chest, back/buttocks, arms, and legs). Despite the observed regional differences in antigen levels, no significant correlation was found between antigen expression and BPDAI/PDAI scores, suggesting that other factors may influence lesion severity and distribution.

## 4. Discussion

Blister formation in AIBD occurs where structural proteins, targeted by autoantibodies, are expressed. Although these target antigens are broadly distributed in the skin and mucosa, blistering occurs preferentially at specific sites rather than being randomly distributed. The role of regional antigen expression as a determinant of this scattered blistering pattern, however, remains largely unexplored. One possible hypothesis is that the abundance of specific target antigens in certain body regions may increase their susceptibility to stronger autoantibody binding and a more robust autoimmune response, thereby localizing blister formation. Conversely, an alternative hypothesis suggests that lower antigen expression in specific areas may make those regions more vulnerable to environmental factors, such as mechanical stress, which could exacerbate blistering. To explore these competing hypotheses, we performed antigen mapping for common AIBD-associated antigens across various body regions of healthy donors, mimicking the physiological condition of antigen expression. This approach will provide insight into whether higher or lower antigen expression correlates with lesion localization, further enhancing our understanding of AIBD pathogenesis. Such findings could also be instrumental for clinicians in predicting disease progression and developing more targeted therapeutic strategies tailored to the specific antigenic profiles of affected body regions.

In this study, we systematically analyzed the clinical scores of BP patients using the validated BPDAI scoring system. We found that skin blisters/erosions and erythematous/urticarial lesions primarily affected arms and legs, findings that were corroborated in a second BP cohort (cohort 2). In our PV cohort, skin lesions were most prevalent on the back, abdomen, and chest, with buccal mucosa being the most commonly affected mucosal surface. PF patients had skin lesions predominantly on the trunk (back, chest, and abdomen), face, and arms. Notably, the scoring systems used do not differentiate between the upper back and lower back or the buttocks.

Next, we examined antigen expression in different anatomical regions by incubating skin and mucosal tissues obtained from healthy body donors with antibodies against AIBD antigens. BP180 expression was significantly higher in arms and palms, while BP230 expression was significantly higher in cheek, buccal/lower labial mucosa, and back compared to conjunctiva. Additionally, a higher expression of integrin β4 was observed in arms. Recently, we reported that laminin β4 is highly expressed in extremities and trunk [17]. Despite these variations, no significant expression differences were seen for other antigens across body sites. Also, we found no correlation between antigen expression (BP180, BP230, and integrin β4) and BPDAI/PDAI clinical scores. These results should be interpreted with caution, as the correlation was estimated from summary protein expression and clinical score data. In this analysis, the units of observation were body sites, and the analysis could be underpowered due to the inclusion of only 10 sites. This suggests that antigen expression does not fully explain the localized distribution of lesions in AIBD. It is important to note that our antigen expression data came from healthy body donors with no history of AIBD. Our results are consistent with previous studies showing minor proteomic differences in primary human keratinocytes across sex, age, and anatomical location [18]. Using mass spectrometry for quantitative proteomic analysis, Sprenger et al. profiled the protein composition of keratinocytes isolated from skin biopsies of healthy donors (*n* = six; three females and three males). Their cohort included individuals across a wide age range (2 to 93 years old) and sampled anatomical sites such as the knee, hip, thigh, arm, and foreskin. In contrast, Ioannides et al. demonstrated variability in the distribution, density, and expression of pemphigus antigens between human skin regions [8]. This discrepancy may be due to methodological variations; e.g., Ioannides et al. used patient sera for antibody titration, whereas we used commercially available antibodies. Their study examined 16 anatomical sites (scalp, face, neck, upper/lower chest, upper/lower back, abdomen, upper arm, forearm, hand, thigh, knee, lower leg, foot, and buccal mucosa) from normal body donors (*n* = three; one female and two males). Using indirect IF with sera containing high titers of PF and PV antibodies, they found regional variations in antigen expression, with PF antigens most prominently expressed in the upper torso and PV antigens most abundant in the buccal mucosa and scalp. While our study also used tissue biopsies from post-mortem donors, our findings differ from those of Ioannides et al. For instance, we did not observe the same degree of regional variation in antigen expression. This discrepancy may arise from several factors, including methodological differences. Ioannides et al. used patient sera for antibody titration, whereas we employed commercially available monoclonal antibodies. Additionally, variations in sample size and anatomical site selection could also contribute to the differing results.

Several skin disorders are characterized by epidermal changes that affect epidermal thickness. A systematic review by Lintzeri et al. found that, aside from the statistically thicker epidermis in the palmoplantar area, which is adapted to withstand mechanical stress and friction, the epidermis in most anatomical sites showed no significant differences in thickness [19]. Similarly, a review by Xu et al. reported that the palms and soles were notably thicker than the head and neck regions [20].

Our study suggests that factors beyond regional variations in antigen expression, such as genetics, epigenetics, microbial, or environmental triggers, may influence where lesions develop in AIBD. We observed that AIBD lesions frequently occur at sites exposed to mechanical stress, such as the extremities in BP and the oral mucosa in PV, likely due to friction-induced immune activation and epidermal disruption. This aligns with findings in experimental epidermolysis bullosa acquisita, where mechanical irritation has been linked to non-healing wounds and chronic inflammation [21]. Epidermal disruption further exacerbates blistering following autoantibody binding and complement activation [22]. Even without epidermal disruption, mechanical irritation via removing the stratum corneum or simple irritation can trigger lesion formation in this model [23].

The role of the human microbiome in skin diseases has recently gained interest [24]. Different body sites harbor distinct microbial communities, and conditions like AIBD disrupt this balance, increasing susceptibility to blistering. A study by Belheouane et al. revealed significant differences in the skin microbiota between BP patients and matched controls, with a loss of protective microbes and an increase in *Staphylococcus aureus*, a pro-inflammatory species [25]. In addition, significant differences in the microbiome composition of non-lesional skin in BP patients compared to the same body sites of age- and sex-matched controls highlighted the skin microbiome as a potential trigger factor in BP. However, the study suggested that the skin microbiota in BP patients is influenced more by disease status than by body site.

Viral infections, particularly herpesviruses, have also been implicated in triggering and exacerbating AIBD, including PV [26], PF [27], and BP [28]. However, the precise link between viral infections and lesion localization remains uncertain.

Additionally, ultraviolet (UV)-induced cell damage has been proposed as a trigger for autoimmune responses, impairing the skin barrier and causing blister formation [29]. For instance, Kano et al. demonstrated that UV-irradiated sites in photoinduced PF exhibited increased autoantibody binding, potentially leading to acantholysis [30]. Similarly, pemphigus lesions often appear in sun-exposed areas, likely due to enhanced antigen expression and UV-induced autoantibody binding [31]. BP lesions may develop on pre-existing psoriatic lesions, possibly triggered by UVB and psoralen plus ultraviolet A (PUVA) exposure [32,33]. BP has also been observed following burns, skin grafts, trauma, surgical wounds, or scabies [34,35,36,37,38,39,40], with the affected areas becoming immunocompromised and susceptible to secondary diseases, such as localized BP, for varying periods ranging from days to decades [41].

Anatomical variations in lesion localization may also be influenced by the immune microenvironment. Tertiary lymphoid structures form in response to immunological needs, generating local immune responses [42]. During cutaneous acquired immune responses, T cells, dendritic cells, and perivascular macrophages form a leukocyte-clustering structure called the inducible skin-associated lymphoid tissue in the dermis [43], serving as a site for antigen presentation [44]. A recent study has shown tertiary lymphoid structures with Dsg-specific B cells in chronic pemphigus blisters [45], suggesting that chronic lesions may be caused by autoantibodies secreted by lesion-resident cells and inflammation mediated by T cell subsets [46]. In vitiligo, anatomically defined subsets of dermal fibroblasts exhibit distinct chemokine expression that recruits CD8^+^ cytotoxic T cells and drives the characteristic depigmentation pattern [47]. Whether similar immune mechanisms contribute to AIBD remains to be explored.

Genetic factors may also influence lesion localization [48]. For instance, certain gene mutations in inflammatory bowel disease, such as those in the *NOD2*/*CARD15* gene, have been associated with Crohn’s disease. Other genetic factors influence whether the disease affects the colon or the small intestine [49]. At the translational level, scalp psoriasis samples showed increased modulation of genes involved in tumor necrosis factor α (TNFα)/ interleukin-17 (IL-17)/IL-22-induced keratinocyte responses compared to that of skin psoriasis samples [50].

In conclusion, our study provides a comprehensive profile of AIBD antigen expression, showing slight regional variations. Lesion localization in AIBD appears to be largely independent of site-specific antigen expression. The interplay between antigen distribution, density, immune response, genetics, and environmental factors is likely to determine blister sites in AIBD patients.

## Data Availability

The data supporting the results of this study are available from the corresponding author, S.E., upon reasonable request.

## Supplementary Materials

The following supporting information can be downloaded at: https://www.mdpi.com/article/10.3390/life15020218/s1, Figure S1. Antibody titration assay using immunofluorescence microscopy. Table S1: List of antibodies used for immunofluorescence staining. Table S2. Frequency and severity of skin involvement in bullous pemphigoid. Table S3. Frequency and severity of erythematous/urticarial lesions in bullous pemphigoid. Table S4. Frequency and severity of mucosal involvement in bullous pemphigoid. Table S5. Frequency and severity of pigmentation in bullous pemphigoid. Table S6. Frequency and severity of mucosal blisters/erosions in PV. Table S7. Frequency and severity of skin and scalp involvement in PV and PF.

## Abbreviations

Autoimmune blistering diseases (AIBDs); basement membrane zone (BMZ); bullous pemphigoid (BP); Bullous Pemphigoid Disease Activity Index (BPDAI); desmoglein (Dsg); epidermal thickness (ET); Pemphigus Disease Area Index (PDAI); pemphigus vulgaris (PV); pemphigus foliaceus (PF).

## References

[1] Zeng F.A.P., Murrell D.F. (2021). State-of-the-art review of human autoimmune blistering diseases (AIBD). Vet. Dermatol..

[2] Emtenani S., Hertl M., Schmidt E., Hudemann C. (2023). Mouse models of pemphigus: Valuable tools to investigate pathomechanisms and novel therapeutic interventions. Front. Immunol..

[3] Schmidt E., Kasperkiewicz M., Joly P. (2019). Pemphigus. Lancet.

[4] Schmidt E., Zillikens D. (2013). Pemphigoid diseases. Lancet.

[5] van Beek N., Weidinger A., Schneider S.W., Kleinheinz A., Glaser R., Holtsche M.M., von Georg A., Hammers C.M., Hubner F., Lima A.L. (2021). Incidence of pemphigoid diseases in Northern Germany in 2016—First data from the Schleswig-Holstein Registry of Autoimmune Bullous Diseases. J. Eur. Acad. Dermatol. Venereol..

[6] Holtsche M.M., Boch K., Schmidt E. (2023). Autoimmune bullous dermatoses. J. Dtsch. Dermatol. Ges..

[7] Du G., Patzelt S., van Beek N., Schmidt E. (2022). Mucous membrane pemphigoid. Autoimmun. Rev..

[8] Ioannides D., Hytiroglou P., Phelps R.G., Bystryn J.C. (1991). Regional variation in the expression of pemphigus foliaceus, pemphigus erythematosus, and pemphigus vulgaris antigens in human skin. J. Investig. Dermatol..

[9] Borradori L., Van Beek N., Feliciani C., Tedbirt B., Antiga E., Bergman R., Bockle B.C., Caproni M., Caux F., Chandran N.S. (2022). Updated S2 K guidelines for the management of bullous pemphigoid initiated by the European Academy of Dermatology and Venereology (EADV). J. Eur. Acad. Dermatol. Venereol..

[10] Schmidt E., Goebeler M., Hertl M., Sardy M., Sitaru C., Eming R., Hofmann S.C., Hunzelmann N., Kern J.S., Kramer H. (2015). S2k guideline for the diagnosis of pemphigus vulgaris/foliaceus and bullous pemphigoid. J. Dtsch. Dermatol. Ges..

[11] Murrell D.F., Daniel B.S., Joly P., Borradori L., Amagai M., Hashimoto T., Caux F., Marinovic B., Sinha A.A., Hertl M. (2012). Definitions and outcome measures for bullous pemphigoid: Recommendations by an international panel of experts. J. Am. Acad. Dermatol..

[12] Joly P., Horvath B., Patsatsi A., Uzun S., Bech R., Beissert S., Bergman R., Bernard P., Borradori L., Caproni M. (2020). Updated S2K guidelines on the management of pemphigus vulgaris and foliaceus initiated by the european academy of dermatology and venereology (EADV). J. Eur. Acad. Dermatol. Venereol..

[13] Murrell D.F., Dick S., Ahmed A.R., Amagai M., Barnadas M.A., Borradori L., Bystryn J.C., Cianchini G., Diaz L., Fivenson D. (2008). Consensus statement on definitions of disease, end points, and therapeutic response for pemphigus. J. Am. Acad. Dermatol..

[14] Holm S. (1979). A Simple Sequentially Rejective Multiple Test Procedure. Scand. J. Stat..

[15] Cochran W.G. (1950). The comparison of percentages in matched samples. Biometrika.

[16] Hothorn T., Bretz F., Westfall P. (2008). Simultaneous inference in general parametric models. Biom. J..

[17] Goletz S., Pigors M., Lari T.R., Hammers C.M., Wang Y., Emtenani S., Aumailley M., Holtsche M.M., Stang F.H., Weyers I. (2024). Laminin beta4 is a constituent of the cutaneous basement membrane zone and additional autoantigen of anti-p200 pemphigoid. J. Am. Acad. Dermatol..

[18] Sprenger A., Weber S., Zarai M., Engelke R., Nascimento J.M., Gretzmeier C., Hilpert M., Boerries M., Has C., Busch H. (2013). Consistency of the proteome in primary human keratinocytes with respect to gender, age, and skin localization. Mol. Cell. Proteom..

[19] Lintzeri D.A., Karimian N., Blume-Peytavi U., Kottner J. (2022). Epidermal thickness in healthy humans: A systematic review and meta-analysis. J. Eur. Acad. Dermatol. Venereol..

[20] Xu H., Fonseca M., Wolner Z., Chung E., Wu X., Geller S., Dusza S.W., DeRosa A.P., Marghoob A.A., Busam K.J. (2017). Reference values for skin microanatomy: A systematic review and meta-analysis of ex vivo studies. J. Am. Acad. Dermatol..

[21] Niebuhr M., Bieber K., Banczyk D., Maass S., Klein S., Becker M., Ludwig R., Zillikens D., Westermann J., Kalies K. (2020). Epidermal Damage Induces Th1 Polarization and Defines the Site of Inflammation in Murine Epidermolysis Bullosa Acquisita. J. Investig. Dermatol..

[22] Pinkus H. (1951). Examination of the epidermis by the strip method of removing horny layers. I. Observations on thickness of the horny layer, and on mitotic activity after stripping. J. Investig. Dermatol..

[23] Hundt J.E., Iwata H., Pieper M., Pfundl R., Bieber K., Zillikens D., Konig P., Ludwig R.J. (2020). Visualization of autoantibodies and neutrophils in vivo identifies novel checkpoints in autoantibody-induced tissue injury. Sci. Rep..

[24] Carmona-Cruz S., Orozco-Covarrubias L., Saez-de-Ocariz M. (2022). The Human Skin Microbiome in Selected Cutaneous Diseases. Front. Cell. Infect. Microbiol..

[25] Belheouane M., Hermes B.M., Van Beek N., Benoit S., Bernard P., Drenovska K., Gerdes S., Glaser R., Goebeler M., Gunther C. (2023). Characterization of the skin microbiota in bullous pemphigoid patients and controls reveals novel microbial indicators of disease. J. Adv. Res..

[26] Tufano M.A., Baroni A., Buommino E., Ruocco E., Lombardi M.L., Ruocco V. (1999). Detection of herpesvirus DNA in peripheral blood mononuclear cells and skin lesions of patients with pemphigus by polymerase chain reaction. Br. J. Dermatol..

[27] Fernandes N.C., Rampinelli H., Souza L.M., Guimaraes M. (2017). Refractory pemphigus foliaceus associated with herpesvirus infection: Case report. Rev. Inst. Med. Trop..

[28] Jang H., Jin Y.J., Yoon C.H., Kim C.W., Kim L. (2018). Bullous pemphigoid associated with chronic hepatitis C virus infection in a hepatitis B virus endemic area: A case report. Medicine.

[29] Sanchez-Palacios C., Chan L.S. (2004). Development of pemphigus herpetiformis in a patient with psoriasis receiving UV-light treatment. J. Cutan. Pathol..

[30] Kano Y., Shimosegawa M., Mizukawa Y., Shiohara T. (2000). Pemphigus foliaceus induced by exposure to sunlight. Report of a case and analysis of photochallenge-induced lesions. Dermatology.

[31] Safadi M.G., Turowski M., Murray T., Zahner S., Aronson I. (2021). Pemphigus vulgaris and foliaceus localized to the nose: Report of 2 cases. JAAD Case Rep..

[32] George P.M. (1995). Bullous pemphigoid possibly induced by psoralen plus ultraviolet A therapy. Photodermatol. Photoimmunol. Photomed..

[33] Wilczek A., Sticherling M. (2006). Concomitant psoriasis and bullous pemphigoid: Coincidence or pathogenic relationship?. Int. J. Dermatol..

[34] Massa M.C., Freeark R.J., Kang J.S. (1996). Localized bullous pemphigoid occurring in a surgical wound. Dermatol. Nurs..

[35] Danescu S., Chiorean R., Macovei V., Sitaru C., Baican A. (2016). Role of physical factors in the pathogenesis of bullous pemphigoid: Case report series and a comprehensive review of the published work. J. Dermatol..

[36] Lo Schiavo A., Caccavale S., Alfano R., Gambardella A., Cozzi R. (2014). Bullous pemphigoid initially localized around the surgical wound of an arthroprothesis for coxarthrosis. Int. J. Dermatol..

[37] Neri I., Antonucci V.A., Balestri R., Tengattini V., Iozzo I., Bardazzi F. (2013). Bullous pemphigoid appearing both on thermal burn scars and split-thickness skin graft donor sites. J. Dtsch. Dermatol. Ges..

[38] Ghura H.S., Johnston G.A., Milligan A. (2001). Development of a bullous pemphigoid after split-skin grafting. Br. J. Plast. Surg..

[39] Moro F., Fania L., Sinagra J.L.M., Salemme A., Di Zenzo G. (2020). Bullous Pemphigoid: Trigger and Predisposing Factors. Biomolecules.

[40] Konishi N., Suzuki K., Tokura Y., Hashimoto T., Takigawa M. (2000). Bullous eruption associated with scabies: Evidence for scabetic induction of true bullous pemphigoid. Acta Derm. Venereol..

[41] Baroni A., Piccolo V., Russo T., Chessa M.A. (2014). Localized bullous pemphigoid occurring on surgical scars: An instance of immunocompromised district. Indian. J. Dermatol. Venereol. Leprol..

[42] Jones G.W., Jones S.A. (2016). Ectopic lymphoid follicles: Inducible centres for generating antigen-specific immune responses within tissues. Immunology.

[43] Honda T., Egawa G., Kabashima K. (2019). Antigen presentation and adaptive immune responses in skin. Int. Immunol..

[44] Kabashima K., Honda T., Ginhoux F., Egawa G. (2019). The immunological anatomy of the skin. Nat. Rev. Immunol..

[45] Han D., Lee A.Y., Kim T., Choi J.Y., Cho M.Y., Song A., Kim C., Shim J.H., Kim H.J., Kim H. (2023). Microenvironmental network of clonal CXCL13+CD4+ T cells and Tregs in pemphigus chronic blisters. J. Clin. Investig..

[46] Xu C., Zhang T., Wang H., Zhu L., Ruan Y., Huang Z., Wang J., Zhu H., Huang C., Pan M. (2023). Integrative single-cell analysis reveals distinct adaptive immune signatures in the cutaneous lesions of pemphigus. J. Autoimmun..

[47] Wang S., Lu M., Zhao Z., Peng X., Li L., Cheng C., Fang M., Xia Y., Liu Y. (2021). Plasma levels of D-dimer and fibrin degradation products correlate with bullous pemphigoid severity: A cross-sectional study. Sci. Rep..

[48] Olbrich M., Kunstner A., Witte M., Busch H., Fahnrich A. (2019). Genetics and Omics Analysis of Autoimmune Skin Blistering Diseases. Front. Immunol..

[49] Zhang Y.Z., Li Y.Y. (2014). Inflammatory bowel disease: Pathogenesis. World J. Gastroenterol..

[50] Ruano J., Suarez-Farinas M., Shemer A., Oliva M., Guttman-Yassky E., Krueger J.G. (2016). Molecular and Cellular Profiling of Scalp Psoriasis Reveals Differences and Similarities Compared to Skin Psoriasis. PLoS ONE.

