# Supplementary material for: Localization of Lesions in Autoimmune Blistering Diseases Is Independent of Site-Specific Target Antigen Expression
Source: Life (Basel). 2025 Jan 31;15(2):218. doi: 10.3390/life15020218 (PMC11857079; doi:10.3390/life15020218)
Supplement: Supplementary file 1 [file life-15-00218-s001.zip › life-3392294-supplementary.pdf]

Supplementary Figures

Figure S1. Antibody titration assay using immunofluorescence microscopy

Representative immunofluorescence (IF) images display the last two positive dilutions of each antibody on cheek tissue taken from a body donor. The nucleus was stained blue with DAPI (blue). Scale bars, 100 µm. IF staining demonstrates linear expression of pemphigoid antigens (BP180, BP230, collagen VII, laminin α3, laminin β3, integrin α6, integrin β4, and plectin) at the basement membrane zone, intraepidermal expression of pemphigus antigens (desmoglein (Dsg)1 and Dsg3), and suprabasal expression of cytokeratin 14.

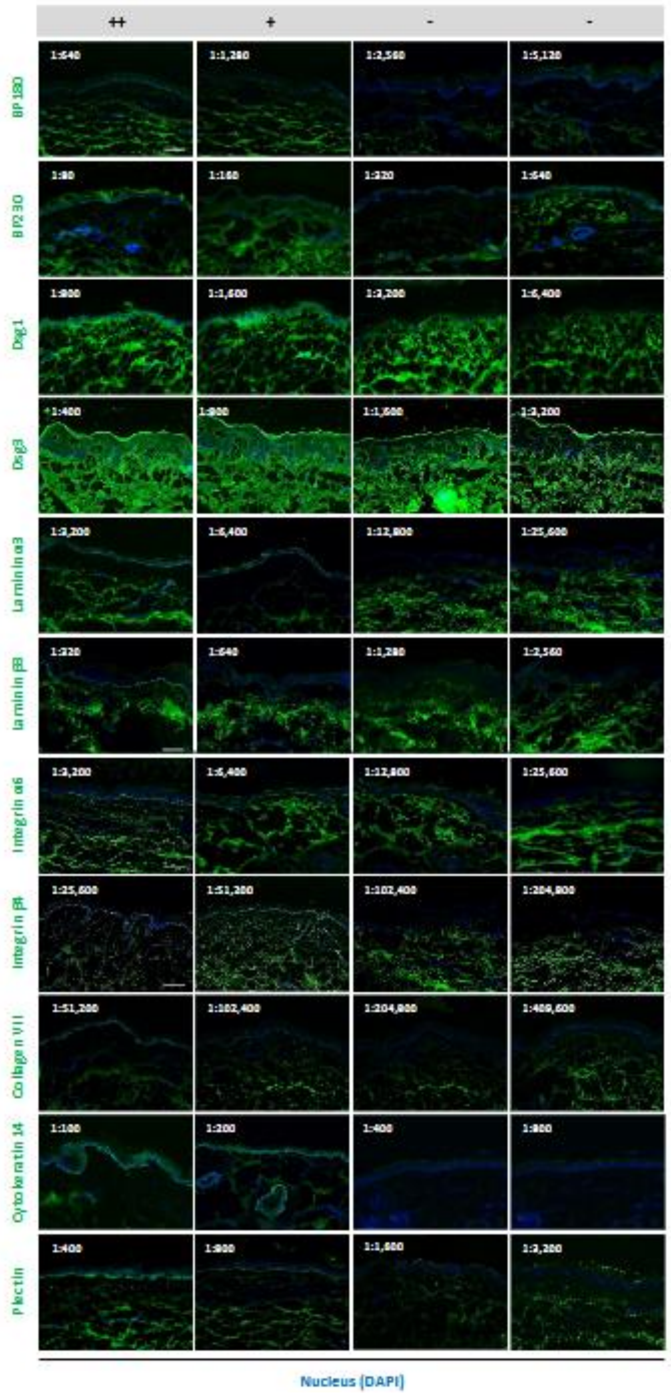
++
+
-
-
BP180
1:640
1:1,280
1:2,560
1:5,120
BP230
1:80
1:160
1:320
1:640
Dsg1
1:800
1:1,600
1:3,200
1:6,400
Dsg3
1:400
1:800
1:1,600
1:3,200
Laminin α3
1:3,200
1:6,400
1:12,800
1:25,600
laminin β3
1:320
1:640
1:1,280
1:2,560
Integrin α6
1:3,200
1:6,400
1:12,800
1:25,600
Integrin β4
1:25,600
1:51,200
1:102,400
1:204,800
Collagen VII
1:51,200
1:102,400
1:204,800
1:409,600
Cytokeratin 14
1:100
1:200
1:400
1:800
Plectin
1:400
1:800
1:1,600
1:3,200
Nucleus (DAPI)

Supplementary Tables

Table S1: List of antibodies used for immunofluorescence staining

Name	Reference	Supplier
**Primary antibodies**		
BP230 (BPAG1)	NU-01-BP1 (Clone: 279)	COSMO BIO CO., LTD., Tokyo, Japan
Collagen VII	C6805 (Clone LH7.2)	Sigma-Aldrich, Darmstadt, Germany
Collagen XVII (BP180)	MM-0065-P (Clone NC16a-3)	MEDIMABS, Montreal (Quebec), Canada
Desmoglein 1	Clone AK2264	Euroimmun, Lübeck, Germany
Desmoglein 3	Clone AK47	Euroimmun, Lübeck, Germany
Integrin α6	sc-13542 (Clone BQ16)	Santa Cruz Biotechnology, INC., Texas, USA
Integrin β4	sc-13543 (Clone A9)	Santa Cruz Biotechnology, INC., Texas, USA
Keratin pan type II	61006 (Clone Ks pan1-8)	Progen, Heidelberg, Germany
Laminin α3	MAB2144 (Clone P3H9-2)	R&D Systems, Minneapolis, USA
Laminin β3	Sc-73519 (Clone 6F12)	Santa Cruz Biotechnology, INC., Texas, USA
Plectin	PA5-56292	Thermo Fisher Scientific, Dreieich, Germany
Mouse IgG1 isotype control	Clone MG-45	BioLegend GmbH, Fell, Germany
Mouse IgG2a isotype control	MOPC-173	BioLegend GmbH, Fell, Germany
**Secondary antibodies**		
Alexa Fluor 488-AffiniPure Goat Anti-Human IgG (H+L)	109-545-088	Jackson ImmunoResearch, Cambridgeshire, UK
Anti-Guinea Pig IgG-FITC	F6261	Sigma-Aldrich, Darmstadt, Germany
Goat anti-Mouse IgG (H+L) Highly Cross-Adsorbed Secondary Antibody, Alexa Fluor Plus 488	A32723	Thermofisher Scientific, Dreieich, Germany

Table S2. Frequency and severity of skin involvement in bullous pemphigoid

Body site	Cohort 1 (n = 65)		Cohort 2 (n = 119)	
	Score 1-3	Score > 3	Score 1-3	Score > 3
Head	18.5%	1.5%	25.0%	2. 8%
Neck	16.9%	1.5%	18.1%	0.7%
Chest	33.8%	6.1%	46.5%	2.8%
Arms	53.8%	13.8%	77.1%	34.0%
Hands	35.4%	6.1%	50.7%	22.9%
Abdomen	41.5%	10.8%	43.1%	13.2%
Genital	7.7%	1.5%	9.0%	3.5%
Back/buttocks	40.0%	9.2%	52.1%	16. 7%
Legs	50.8%	15.4%	75.7%	41. 7%
Feet	36.9%	15.4%	50.7%	31.9%

Table S3. Frequency and severity of erythematous/urticarial lesions in bullous pemphigoid

Body site	Cohort 1 (n = 65)		Cohort 2 (n = 119)	
	Score 1-3	Score > 3	Score 1-3	Score > 3
Head	6.1%	0.0%	12.5%	0.0%
Neck	10.8%	0.0%	23.6%	0.7%
Chest	30.8%	3.1%	61.1%	2.1%
Arms	50.8%	3.1%	84.7%	11.1%
Hands	23.1%	1.5%	40.3%	1.4%
Abdomen	30.8%	1.5%	65.8%	4.2%
Genital	1.5%	0.0%	7.6%	0.0%
Back/buttocks	40.0%	4.6%	72.2%	4.2%
Legs	44.6%	4.6%	77.8%	19.4%
Feet	26.1%	3.1%	36.8%	1.4%

Table S4. Frequency and severity of mucosal involvement in bullous pemphigoid

Body site	Cohort 1 (n = 65)		Cohort 2 (n = 119)	
	Score 1-2	Score > 2	Score 1-2	Score > 2
Eyes	0.00%	0.00%	0.69%	0.00%
Nose	1.54%	0.00%	1.39%	0.00%
Buccal mucosa	7.69%	0.00%	4.17%	0.00%
Hard palate	1.54%	0.00%	3.47%	0.00%
Soft palate	4.62%	0.00%	2.08%	0.00%
Upper gingiva	3.08	0.00%	1.39%	0.00%
Lower gingiva	1.54%	0.00%	1.39%	0.00%
Tongue	1.54%	0.00%	1.39%	0.00%
Mouth floor	0.00%	0.00%	0.69%	0.00%
Posterior pharynx	0.00%	0.00%	0.00%	0.00%
Angogenital	1.54%	0.00%	2.08%	0.00%

Table S5. Frequency and severity of pigmentation in bullous pemphigoid

Body site	BP cohort 1 (n = 65)	
	Score 1-3	Score > 3
Head	3.08%	0.0%
Neck	3.08%	0.0%
Chest	16.9%	0.0%
Hands	12.3%	0.0%
Abdomen	21.5%	0.0%
Genital	1.5%	0.0%
Back/Buttocks	29.2%	0.0%
Feet	15.4%	0.0%
Legs	36.9%	0.0%
Arms	36.9%	0.0%

Table S6. Frequency and severity of mucosal blisters/erosions in PV

Body site	PV cohort (n = 67)	
	Score 1-3	Score > 3
Eyes	0.0%	0.0%
Nose	37.3%	7.5%
Buccal mucosa	53.7%	20.9%
Hard palate	22.4%	10.4%
Soft palate	22.4%	9.0%
Upper gingiva	25.8%	10.4%
Lower gingiva	28.3%	11.9%
Tongue	17.9%	6.0%
Mouth floor	17.9%	4.5%
Labial mucosa	23.9%	10.4%
Posterior pharynx	6.0%	4.5%
Anogenital	17.9%	0.0%

Table S7. Frequency and severity of skin and scalp involvement in PV and PF

Body site	PV cohort (n = 67)		PF cohort (n = 20)	
	Score 1-3	Score > 3	Score 1-3	Score > 3
Ears	11.9%	1.5%	20.0%	0.0%
Nose	13.4%	1.5%	40.0%	0.0%
Rest of the face	22.4%	0.0%	65.0%	0.0%
Neck	19.4%	3.0%	35.0%	0.0%
Chest	37.3%	4.5%	55.0%	15.0%
Abdomen	34.3%	8.7%	55.0%	10.0%
Back/Buttocks	38.8%	8.7%	75.0%	15.0%
Arms	25.4%	1.5%	55.0%	15.0%
Hands	7.5%	1.5%	5.0%	0.0%
Legs	20.9%	3.0%	45.0%	5.0%
Feet	7.5%	1.5%	1.5%	0.0%
Genital	13.4%	1.5%	1.5%	0.0%
Scalp	17.9%	8.7%	63.2%	36.8%
